# Factors associated with late presentation of patients with chronic kidney disease in nephrology consultation in Cameroon-a descriptive cross-sectional study

**DOI:** 10.1080/0886022X.2019.1595644

**Published:** 2019-05-20

**Authors:** Halle Marie Patrice, Nyongbella Joiven, Fouda Hermine, Balepna Jean Yves, Kaze Folefack François, Ashuntantang Enow Gloria

**Affiliations:** a Faculty of Medicine and Pharmaceutical Sciences, University of Douala, Douala, Cameroon;; b Department of Internal Medicine, Douala General Hospital, Douala, Cameroon;; c Faculty of Medicine and Biomedical Sciences, University of Yaoundé I, Douala, Cameroon;; d Department of Internal Medicine, Douala Laquintinie Hospital, Douala, Cameroon;; e Faculty of Medicine and Biomedical sciences, Yaoundé General Hospital Cameroon, Douala, Cameroon

**Keywords:** Late referral, chronic kidney disease, nephrology, Douala

## Abstract

**Background:** Late presentation (LP) of chronic kidney disease (CKD) patients to nephrologist is a serious problem worldwide with persistent high prevalence despite known benefits of early nephrology care.

**Objective**: Determine the prevalence and factors associated with LP of CKD patients to nephrologists in Cameroon.

**Methods:** A cross-sectional study from October 2015 to May 2016 at the nephrology units of the Douala General and Laquintinie hospitals, including all consenting incident CKD patients. Data collected were: socio-demographic, search of CKD diagnostic criteria during prior follow up, therapeutic itinerary, clinical and biological parameters at presentation, knowledge on CKD and attitude towards dialysis. LP was defined as eGFR < 30 ml/min/1.73 m^2^. It was physician-related whenever no CKD screening was done in the presence of risk factor or no referral to nephrologists at early stages; patient-related whenever patients did not have recourse to hospital care while symptomatic or disrespected a referral decision. *p* value <.05.

**Results:** We included 130 patients, mean age 53.10 ± 14.66 years, 60.77% males, 58.70% were referred by internal medicine physicians and 10% had recourse to complementary and alternative medicine (CAM). At presentation, 70.80% were symptomatic, 53% had CKD stage five, 86.12% were poorly graded on knowledge and 49% had a negative attitude towards dialysis. The prevalence of LP was 73.90%, 50% was physician-related, 44.79% patient-related and 5.21% both. Being accompanied (*p* = .038), a low level of education (*p* = .025) and recourse to CAM (*p* = .008) were associated with LP.

**Conclusion:** LP is high in Cameroon, attributed to physician’s practical attitudes and patient’s socio-cultural behaviors and economic conditions.

## Introduction

Chronic kidney disease (CKD) is a growing health problem with a high economic cost to health systems [1]. It’s global prevalence varies from 11 to 13% with a reported incidence rate more than doubling in many countries over the past two decades, and alarming forecasts for the years to come [2,3]. The picture in low income countries such as those of sub-Saharan Africa (SSA) is not well known due to lack of national registries [4,5]. However, CKD is a serious burden given the reported high prevalence of risk factors such as hypertension, diabetes mellitus, infectious diseases (HIV, hepatitis B and C), low socioeconomic status and the relatively poor access to preventive measures [4,5].

CKD is known to progress silently in five stages to End Stage Kidney Disease (ESKD) which carries a high morbidity, mortality and healthcare cost [6,7]. If actively searched with simple measures, early detection of CKD will allow the implementation of strategies to delay progression of the disease to end stage [6]. However, most patients are not benefiting from these preventives measures, because they are referred late, if referred at all to the nephrologist [8,9]. There is no universally accepted definition for late presentation [10], but most authors use the time from first consultation to start of dialysis which varies among authors from 3 to 12 months while a few authors with the intention of broadening the definition have used the level of GFR/CKD stage on admission to define late presentation. Definitions based on time-to-start of dialysis presupposes that general practitioners, internists and other specialists are able to predict when dialysis will be necessary in any given patient – a difficult skill even for trained nephrologists. Also these definitions might underestimate the impact of late presentation (LP) since it ignores the large group of patients with impaired renal function, in whom the intervention of a nephrologist can be of use to slow down progression and treat secondary complications, as it is well established that the majority of CKD stage 3 patients will die because of cardiovascular diseases even before they will reach End Stage Renal Disease (ESRD) [10].

Despite efforts into sensitization of healthcare professionals, promulgation of guidelines, and the known deleterious consequences of LP of patients for specialist care, the prevalence of LP remains high ranging from 30–82% worldwide [10–17]. Therefore there is a need for more proactive preventive measures based on a thorough analysis of associated factors which are varied [10,18]. Disease related factors such as irreversible acute kidney injury, superimposed acute on CKD, silent and unnoticed disease progression generally leads to unavoidable LP and it accounts for less than 15–20% of cases in the literature [11,12]. Patient related factors such as older age, co-morbidities, belonging to a minority group, lack of health insurance and unemployment are associated with LP [11,17,19–21]. Mechanisms underlying these factors include: knowledge deficits (limited awareness of CKD [22], and understanding of the dialysis process [9,10,23]), negative attitudes (denial of the progressing disease state and refusal to accept the need for dialysis [10]), and economic concerns [18]. Studies have reported that the lack of communication between primary care physicians and nephrologists contributes to late referral (LR) and that LR occurred more commonly by internal medicine physicians and other specialists than by general practice physicians [20,21,24,25]. The type of healthcare system, the density of nephrologists within a given geographic area, and geographic distance to nephrologists are other factors contributing to LP [18,26,27].

In Sub Saharan Africa (SSA), LP is a serious problem with one of the highest prevalence [16], and data regarding associated factors are scarce. A study in South Africa by Madala et al. reported that the presence of comorbidities such as Hypertension and HIV were strongly associated with a low eGFR at presentation [28]. In Cameroon a country of SSA, Halle et al. reported a prevalence of LR of 82.8%, with high hospitalization and emergency dialysis rates on a temporary catheter [16]. At the time of that study there was a scarcity of kidney specialists and nephrology services (four nephrologists and two dialysis centres in two regions of the country). In the recent decade, much amelioration has been done and in 2015 the country counted 11 dialysis centers in eight regions and 12 nephrologists. Despite these ameliorations, LR rates is still high [29], thus remaining a serious problem in our setting. We therefore aimed to identify factors associated with LP of CKD patients to the nephrologist.

## Material and methods

### Study settings

This was a descriptive cross-sectional study from October 2015 to May 2016 conducted in the nephrology services of the Douala General hospital (DGH) and the Douala Laquintinie hospital (DLH) in the littoral region of Cameroon. DGH is a 320 bedded tertiary public institution which serves as the main referral hospitals for patients with kidney diseases in the region whose population at the end of the year 2015 was more than three million [30]. Its hemodialysis unit is the unique public dialysis center of the region with a medical staff made up of two nephrologists and one General practitioner. Outpatient consultations are done from Mondays to Fridays, and the service has about 360 monthly consultations with an average of 90 new patients each month. The DLH is a second category hospital in the region with no dialysis center at the time of this study but has a nephrologist who consults on Mondays and Wednesdays with an average of 25 new patients per month. A medical file is created for all outpatients at the secretariat, while patients’ entering through the emergency are managed as hospitalized patients and after being discharged a file is created in the unit for follow up. The study received administrative authorizations from both hospitals and an ethical approval from the institutional research board of the Douala University.

### Data collection

**Figure 1.**
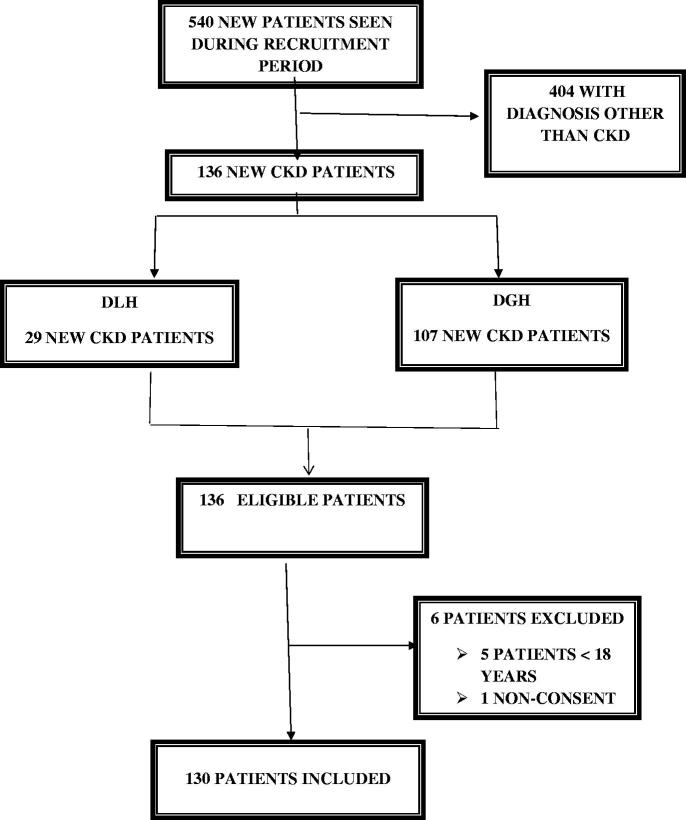
Flow chart representing the selection and inclusion of participants in the study.

We enrolled all consenting incident patients with CKD aged 18 years and above, that were seen in both hospitals during the data collection period. Patients that had acute kidney injury as well as patients with a diagnosis other than kidney disease were not included in our study (Figure 1). Data were collected by a final year medical student using a pretested questionnaire. Eligible patients were identified and data collected were: socio-demographics, (age, gender, marital status, level of education, source of funding, monthly income, residence and presence of an accompanying person), Search of CKD diagnostic criteria during patients prior follow up (kidney abnormalities such as elevated creatinine, albuminuria, abnormalities in urine sediment and kidney ultrasound), therapeutic itinerary of patients (different therapies taken prior to presentation to the nephrologist such as modern auto medication, modern medicine, complementary and alternative medicine (CAM)), hospital category, the specialty of the treating physician and their attitude towards observed kidney abnormalities, reasons that delayed presentation such as patient’s disrespect of referral decision, delay in seeking hospital care, and physician’s failure to screen for CKD and timely refer patients. Clinical data at first consultation of the nephrologist such as: referring physician, referring hospital category, motive of consultation, signs and symptoms, comorbidities, baseline nephropathy and laboratory parameters (serum creatinine and dipstick proteinuria). Data on knowledge and attitude of patients toward CKD. Knowledge was assessed using a series of seven questions (Table 1).

**Table 1.** Assessment of patient’s general knowledge on CKD.

Q01	How many healthy kidney(s) does a person need to lead a normal life?
	1 = One, 2 = Two, 3 = Three, 4 = Four, 5= I don’t know, 6 = others/precise…….
Q02	What is the function of a kidney in a human body? 1 = To break down food, 2 = To produce substances that break down fats, 3 = To filter waste products in the blood, 4 = I don’t know, 5 = Others/precise…………
Q03	What can cause kidney disease? 1 = High blood pressure, 2 = Drinking alcohol, 3= Diabetes, 4 = Inadequate sleep, 5 = Inherited condition, 6 = All of the above, 7 = I don’t know, 8 = others/precise………..
Q04	What are the symptoms of early kidney disease that might progress to kidney failure? 1 = Bubbles in the urine, 2 = Back pain, 3 = Blood in the urine, 4 = Can present without any symptoms/ complaints, 5 = All of the above, 6 = I don’t know, 7 = Others/precise………..
Q05	Which of the following statement about kidney disease is INCORRECT:
	1 = Kidney disease can be prevented. 2 = Kidney disease can be cured with medications. 3 = A person is said to have kidney disease when he/she needs dialysis. 4 = None of the above 5 = I don’t know
Q06	Where can dialysis treatment be carried out? 1= In a dialysis center or at home. 2 = Only in a dialysis center. 3 = Only at home. 4 = I don’t know.
Q07	7. What is the best medical treatment for End Stage Kidney Failure?
	1 = Medication. 2 = Dialysis. 3 = Kidney transplant. 4 = I don’t know.

### Calculations and definitions

Glomerular filtration rate was estimated (e GFR) using the four-variable, abbreviated Modification of Diet in Renal Disease (MDRD) formula [26] and CKD was classified in five stages based on the KDIGO’s classification [4] as followed: G1 *=* GFR > 90 mL/min/1.73 m^3^, G2 = GFR 60–89 mL/min/1.73 m^3^, G3A = GFR 45–59 mL/min/1.73 m^3^, G3B = GFR 30–44 mL/min/1.73 m^3^, G4 = GFR 15–29 mL/min/1.73 m^3^, G5 = GFR < 15 mL/min/1.73 m^3^.

Albuminuria was categorized based on the number of crosses on the urinary dipstick; A_1_=negative to trace, A_2_= +1, A_3_= ≥+2.

Late presentation was considered if a patient presented with CKD stages 4 or 5 (e GFR < 30 mL/min/1.73 m^3^) for the first time, and Early presentation if patient presented with CKD stages 1–3 (eGFR ≥ 30 mL/min/1.73 m^3^).

Patient-related delay was considered if patients presented late due to non- recourse to biomedicine at the beginning of symptoms or disrespect of a referral decision, and Physician-related if during the patient’s follow-up, no CKD screening was done in the presence of a risk factor (or signs of complications) or the physician did not refer the patient in an early stage of the disease.

**Knowledge:** Knowledge was graded as poor, average and good, respectively for participants who had 0–3, 4–5 and 6–7 correct answers out of the seven questions used to assess knowledge.

### Statistical analysis

Data was analyzed using the software STATA version 11 (College Station, TX, USA). Categorical variables were represented as frequencies, percentages and ratios, and compared using the Fischer’s exact test. Quantitative variables were represented as means or medians and compared using the Wilcoxon Mann–Whitney’s test. To identify factors associated with LP socio-demographic and clinically relevant variables from univariate analysis (based on a *p* < .20) were introduced in multivariate logistic regression models in order to measure their association with LP. The odds ratio and its 95% confidence interval were determined for each variable and a *p* < .05 was considered statistically significant.

## Results

**Figure 2.**
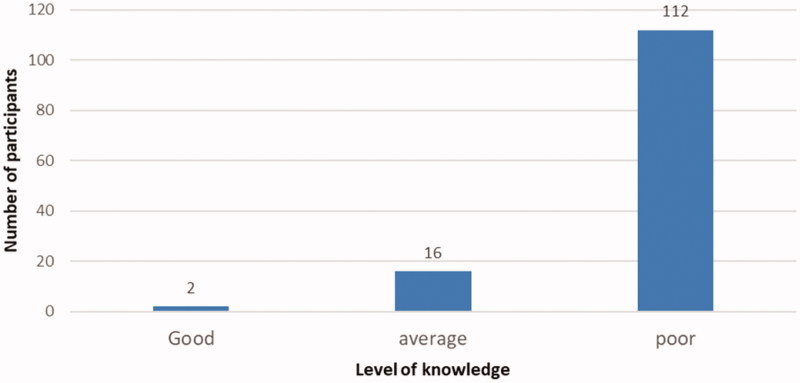
Distribution of level of knowledge on CKD in the study population.

We included a total of 130 patients of which 78.70% (102/130) were from the DGH (Table 2). The mean age was 53.10 ± 14.66 year with 60.77% (79/130) being male. Overall, patients that presented late were significantly younger than their counterparts (*p* = .03), however passed the age of 60 years patients were more likely to present late than early (*p* = .03). A total of 90% (117/130) had no health insurance. Hypertension 70.77% (92/130) and diabetes mellitus 41.54% (54/130) were the most frequent comorbidities. The median GFR was 13.0 mL/min/1.73 m^2^ (range: 00–108 mL/min/1.73 m^2^) with 53.08% (69/130) participants at CKD stage five (Table 2). We found that 86.15% (112/130) of participants had a poor knowledge on CKD (Figure 2). Patients were referred mostly by internal medicine physicians 58.68% (71/121) and general practitioners 38.84% (37/121). A total of 40.5% (49/121) participants came from a 4th category hospital and 10% (13/130) of patients had recourse to complementary and alternative medicine (CAM) before first nephrology consultation (Table 2).

**Table 2.** Baseline characteristics of the study population.

Variable	EP *N* = 34	LP *N* = 96	Total *N* = 130	*p* value
Age (mean)	67.61 ± 13.54	51.50 ± 14.76	53.10 ± 14.66	.03
Age groups *n*(%)	01 (02.94)	01 (01.04)	02 (01.54)	.45
[20]	03 (08.82)	22 (22.92)	25 (19.23)	.05
[20;40]	12 (35.29)	41 (42.71)	53 (40.77)	.29
[40;60]	18 (52.94)	32 (33.33)	50 (38.46)	.03
[60]				
Sex *n*(%)				
Male	23 (67.65)	56 (58.33)	79 (60.77)	
Female	11 (32.35)	40 (41.67)	51 (39.23)	.22
Level of education *n*(%)				
None	00 (00.00)	04 (04.17)	04 (03.08)	.29
Primary	14 (41.18)	29 (30.21)	43 (33.08)	.16
Secondary	11 (32.35)	40 (41.67)	51 (39.23)	.22
University	09 (26.47)	23 (23.96)	32 (24.62)	.46
Source of funding *n*(%)				
Patient	15 (44.12)	33 (34.38)	48 (36.92)	.21
Spouse	01 (02.94)	05 (05.21)	06 (04.62)	.50
Insurance	04 (11.76)	09 (09.38)	13 (10.00)	.45
Family	14 (41.18)	49 (51.04)	63 (48.46)	.21
Residence *n*(%)				
Urban	30 (88.24)	65 (67.71)	95 (73.08)	
Rural	04 (11.76)	31 (32.29)	35 (26.92)	.01
Presence of an accompanying person *n* (%)				
No	20 (58.82)	14 (14.58)	34 (26.15)	
Yes	14 (41.18)	82 (85.42)	96 (73.85)	˂.001
Referred patients				
Yes	31 (92.08)	90 (93.75)	121 (93.08)	
No	03 (08.82)	06 (06.25)	09 (06.92)	
Source of patients				
4th Category	10 (32.26)	39 (43.33)	49 (40.50)	.19
3rd Category	01 (03.23)	10 (11.11)	11 (09.09)	.17
2nd Category	06 (19.35)	17 (18.89)	23 (19.01)	.57
1st Category	09 (29.03)	12 (13.33)	21 (17.36)	.04
Clinic	05 (16.13)	12 (13.33)	17 (14.05)	.45
Referring physician				
General practitioner	13 (41.94)	34 (37.78)	47 (38.84)	.41
Internalmedicine	17 (78.12)	54 (60.00)	71 (58.67)	.25
Surgeon	00 (00.00)	01 (01.11)	01 (00.83)	.74
Others^a^	01 (03.23)	01 (01.11)	02 (01.65)	.71
Comorbidities				
Hypertension	21 (61.76)	71 (73.95)	92 (70.76)	.13
Diabetes	16 (47.05)	38 (40.42)	54 (41.53)	.28
HIV	03 (08.82)	08 (08.51)	11 (08.46)	.58
Gout	04 (11.76)	05 (05.31)	09 (06.92)	.18
Others^b^	06 (17.64)	10 (10.63)	16 (12.31)	.12
Baseline nephropathies				
Diabeticnephropathy	13 (38.24)	29 (30.21)	42 (32.31)	.25
CGN	05 (14.71)	26 (27.08)	31 (23.85)	.10
Hypertensive nephropathy	08 (23.53)	19 (19.79)	27 (20.77)	.40
HIV	02 (05.88)	08 (08.33)	10 (07.69)	.48
Mixed nephropathy	01 (02.94)	05 (05.21)	06 (04.62)	.50
ADPKD	03 (08.82)	01 (01.04)	04 (03.08)	.05
Unknown	01 (02.94)	03 (03.13)	04 (03.08)	.72
Others^c^	01 (02.94)	04 (05.20)	05 (03.85)	.55
Creatinine mg/l (Median)	16.65	71.55	48.6	˂.001
GFR ml/min/1.73 m^2^ (Median)	44	09	13	˂.001

CGN: Chronic glomerulonephritis; ADPKD: Autosomal dominant polycystic kidney disease; GFR: Glomerular Filtration Rate; EP: Early Presentation; LP: Late Presentation.

^a^Occupational physician, public work official. ^b^Hepatitis B, Hepatitis C, Nephrolithiasis, cardiomyopathy, prostate hypertrophy, tuberculosis, and stroke. ^c^Chronic tubulo-interstitial nephritis, Malformation, Obstructive nephropathies, Multiple myeloma.

1st Category: General hospitals and University teaching hospital (CHU), 2nd Category: Central Hospitals (‘L’Hopital Central de Yaounde, L’Hopital Jamot de Yaounde, et L’hopital Laquintinie de Douala’), 3rd Category: Regional hospitals, 4th Category: District hospitals, and all lower level hospitals.

**Figure 3.**
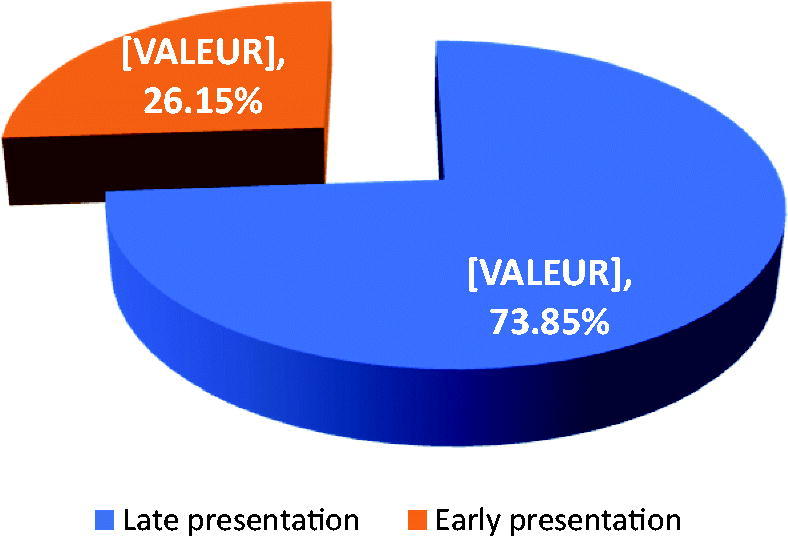
Distribution of the population according to the manner of presentation.

The prevalence of LP was 73.85% (96/130) (Figure 3), and 50% (48/96) was physician related, 44.79% (43/96) patient related and 5.21% (05/96) both (Table 3). The lack of CKD screening 64.15% (34/53) and non-referral to nephrologists in early stages of disease 35.85% (19/53) were the reasons accounting for physician related delays. Failure to seek hospital care in a timely manner 81.25% (39/48) and disrespect of a referral decision 18.75% (09/48) were the reasons for patient related delays (Table 3). In multivariate analysis, being accompanied (*p* = .038), having a level of education below university level (*p* = .025) and the recourse to CAM (*p* = .008) were the patient related factors independently associated with LP. No factor was associated with physician related LP (Table 4).

**Table 3.** Distribution of the study population according to types and related reasons for late presentation.

Types of late presentation	Frequency (*n* = 96) *n* (%)
Physician related delay	**53 (50.00)**
No screening for CKD	34 (64.15)
No referral in early stages	19 (35.85)
Patient related delay	**48 (44.79)**
No recourse to biomedicine	39 (81.25)
Disrespect of referral	09 (18.75)
Both delays	**05 (05.21)**

Bold indicates the major categories of late presentation.

**Table 4.** Multivariate logistic regression analysis of factors associated with late presentation.

Physician-related variables	OR (95% CI)	*p* value
Age >55 years	0.66 (0.29–1.47)	.309
Eodema	1.89 (0.85–4.18)	.117
Specialty of physician		
General practitioner	Ref	
Internal medicine specialist	0.60 (0.23–1.57)	.295
Surgery specialist	1.01 (0.05–21.23)	.993
Others	1.31 (0.06–27.14)	.860
Hospital structure		
Clinic	Ref	
1st Category	2.07 (0.35–12.27)	.425
2nd Category	1.80 (0.34–09.30)	.483
3rd Category	1.77 (0.22–14.34)	.592
4th Category	3.72 (1.84–16.71)	.071
Patient-related variable		
Level of education		
None	Ref	
Primary	0.34 (0.03–3.91)	.388
Secondary	0.23 (0.02–2.68)	.242
Higher	0.56 (0.34–0.93)	**.025**
Know someone with CKD	1.83 (0.43–7.75)	.413
Presence of an accompanying person	2.83 (1.07–7.45)	**.036**
Decision to go for hospital care	0.54 (0.25–1.20)	.132
Recourse to CAM	7.72 (1.70–37.0)	**.008**

CAM: Complementary and Alternative Medicine; CKD: Chronic Kidney Disease. Bold values are statistically significant.

## Discussion

The aim of this study was to determine the factors associated with LP of CKD patients to nephrologists in Douala – Cameroon. We found that 3 patients out of 4 presented late, due to the treating physician (50%), the patient (44.79%) or both (5.21%). Being accompanied at presentation, having a low level of education and the recourse to CAM were the patient-related factors that were associated to LP.

Our participants were relatively young. This is in accordance with previous studies in SSA [16,29,31,32] but contrast results were reported by other studies in developed countries [11,20,33] and this can be explained by the early onset, high prevalence, and severity of risk factors for CKD, including hypertension, diabetes, glomerulonephritis and HIV infection, which are not regularly detected and appropriately managed in our setting [5].

LP of CKD patients to nephrologists have been reported in many regions of the world with remarkable high rates in developing countries, especially in SSA [14,16]. In Cameroon, LP is a serious problem as shown by previous studies [16,29]. Overall, our results seems to agree with the high rates previously found in our setting while contrasting the lower rates seen in developed countries [10,20]. We found a relatively lower rate of 73.8% compared to the 83% obtained by Halle and colleagues in 2009 [16], this slightly lower rate can be accounted for by the net amelioration in the nephrology sector with increase staff and nephrology units across the country in the past years such that more and more physicians and patients are becoming aware of the disease and react more appropriately. However this rate is still very high compared to current rates in other parts of the globe especially developed countries [34]. These persistent high rates in our settings show that both physicians and patients are still not adequately sensitized despite the efforts into awareness [17]. As a consequence, as high as 96% of late referred patient still undergo emergency dialysis on a temporary catheter, with higher rates of hospitalizations and a poor short term survival rate [29].

Physician related delays were due to failure to screen for CKD (64.15%) and failure to refer at an early stage of the disease (35.85%). This finding is supported by the results from Boulware et al. in the United States who found that non-nephrologist physicians were 40% less likely to recognize CKD and recommend referral than nephrologist [35]. The high rates of inability to actively search and timely refer CKD patients in our setting can be explained by the limited knowledge of physician on the nature of CKD and clinical practice guidelines. A study of Choukem et al. in Cameroon reported that 41.20% physicians were unaware of the definition of CKD, only 44% could recognize that CKD had 5 stages, up to 12.70% would still use serum creatinine alone for diagnosis while 21.90% of physicians would refer at late stage [36]. Moreover, in a setting where the number of nephrologists (12 nephrologists mostly found in the big cities of the country at the time of the study) and healthcare facilities to manage the ever growing number of patients with kidney diseases is limited, most patients likely be seen for the first time by a non-nephrologist physician, thus increasing their likelihood to have a physician related delay.

We identified that failure to seek hospital care (81.25%) and disrespect of referral decision (18.75%) were the reasons that led to patient related delay. In our socio cultural setting where the presence of disease or affection is often assimilated with the presence of symptoms [37], the lack of understanding of the silent nature of CKD [37], the huge economic constraints; with medical costs being out of pocket payment due to very low levels of health insurance coverage [38], the low levels of education, and the sociocultural predisposition to seek a comparatively less expensive traditional and complementary medicine [37,38] can justify the high rates of non-recourse to hospitals for care. Referral non adherence on the other hand could be explained by the denial of disease condition, misconception and limited knowledge on the dialysis process creating fear and refusal to accept the need for dialysis, as suggested by a number of authors [23,39,40].

Factors independently associated with patient related LP were, being accompanied at presentation, the recourse to CAM and a level of education below university level. To our knowledge, previous studies did not treat this aspect of the study. The presence of an accompanying person might suggest the fact that being in a deleterious state of health, the patient so much needs moral support from some brethren. Also, an accompanying person could be the source of financial support and so the time lapse between the actual need for hospital care and for the source of finance to judge timely the need for care and provide finances might delay recourse to the hospital. Finally, the patient might be in a state of denial to accept the disease condition and so need someone to push him/her to the fact creating delay in resorting hospital care. The recourse to CAM could be explained by the fact that CAM is relatively cheap for patients facing huge economic constraints becoming therefore an alternative to these patients who might equally be in a state of denial and fear of dialysis [37]. Moreover, CAM might also have a symptomatic effect creating a healing impression in patients giving them hope especially in therapies that have received appraisal. As such patients will consume these therapies as long as they feel better thus lengthening the delay period and will only seek nephrology care when their condition is deleterious [41]. The low levels of education could be explained by the fact that participant with a university level are more cultured on common health problems and will more likely seek hospital care, understand and adhere to physician’s therapeutic decisions than those with a lower level of education [42]. Also, those with higher levels of education might have better economic conditions and thus more likely to seek and comply with hospital care.

Our results are somewhat in accordance with literature reports, however the socio cultural and economic considerations (patients not seeking hospital care on time, low levels of education attainments and understanding of health problems, low income and insurance coverage rates) of our setting as revealed as affecting timing of referral needs a special attention. Also the insufficient knowledge of primary care physicians on existing guidelines regarding timing of nephrology referral, high levels of unawareness of CKD and risk factors in the general public as well as the lack of a national guideline on the management of CKD are important parameters to pay attention to. We therefore recommend the establishment and wide diffusion of national guidelines and educational strategies on the management of CKD targeting both physicians and patients as well as further research initiative to explore the insights of physician factors.

### Limitations and strength of the study

We acknowledge some limitations; provider physicians were not directly addressed hence it was not possible to have an inside on physician factors that could have better explained the high percentage. This was a double centered cross sectional study with actual interviewing of patients thus the shortcomings of retrospective design studies such as missing data were eliminated. We recruited only incident patients during our study period who still had a good memory of their health history.

## Conclusion

This study reveals that in our setting 3 out of every 4 CKD patients, still present late to nephrologist indicating that physicians and patients are still not adequately sensitized on CKD. The practical attitudes of physicians, and the socio-cultural behaviors and economic conditions of patients were revealed as affecting timeliness of presentation. Major emphasis should therefore be laid on physician’s ability to systematically screen and timely refer CKD patients as well as improving patients’ economic conditions and increasing the awareness of CKD in the general public.
